# Unmet need for family planning among married women in Zambia: lessons from the 2018 Demographic and Health Survey

**DOI:** 10.1186/s12905-022-01709-x

**Published:** 2022-04-27

**Authors:** Harriet Namukoko, Rosemary Ndonyo Likwa, Twaambo E. Hamoonga, Million Phiri

**Affiliations:** 1 Population and Demography Unit, Zambia Statistics Agency, Lusaka, Zambia; 2 Department of Population Studies and Global Health, School of Public Health, University of Zambia, Lusaka, Zambia; 3 Department of Population Studies, School of Humanities and Social Sciences, University of Zambia, Lusaka, Zambia; 4 Department of Demography and Population Studies, School of Public Health and Social Sciences, University of Witwatersrand, Johannesburg, South Africa

**Keywords:** Unmet need, Family planning, Determinants, Married women, Zambia, DHS 2018

## Abstract

**Background:**

Unmet need for family planning among married women is still a public health concern in sub-Saharan Africa. In Zambia, one in every five married women had an unmet need for family planning in 2018. Unmet need for family planning has the potential to increase the number of unintended pregnancies and unsafe abortions. These factors can increase the proportion of women of child bearing age, who are at high risk of birth complications. This study was therefore conducted to understand the determinants of unmet need for family planning among married women in Zambia based on recent cross-sectional data.

**Methods:**

The study analysed data extracted from the Zambia Demographic and Health Survey, which was a representative cross-sectional survey conducted in 2018. The analysis was done on a sample of 7598 currently married women aged 15–49 years. Multivariate logistic regression was used to examine determinants of unmet need for family planning in Zambia. The analyses was weighted to account for complex sample design.

**Results:**

Prevalence of unmet need for family planning is still high in Zambia at 20%. Women in the age groups 25–34 and 35–49 were less likely to have total unmet need for family planning (AOR = 0.61; 95% CI 0.47, 0.78) and (AOR = 0.63; 95% CI 0.45, 0.86) respectively, compared with those aged 15–24 years. Age of a woman, parity, household wealth and exposure to media-based family planning messages were found to be significantly associated with unmet need for family planning among married women.

**Conclusion:**

There were significant differences in unmet need for family planning based on a woman's age, number of children ever born, wealth level, and exposure to media-based family planning messaging. Improving access to family planning messages and addressing underlying structural factors that improve the wealth status, particularly among young women, may help to reduce unmet need for family planning in Zambia.

## Background

Promotion of contraception is important in the reduction of fertility and affects maternal morbidity and mortality in most developing countries [1]. Globally, 12% of women in marriage or in-union were estimated to have unmet need for family planning by 2017; this meant that twelve out of every 100 married women who wanted to stop or delay giving birth could not do so because they were not using any method of contraception [1, 2]. Among the regions in the world, sub-Saharan Africa has the highest unmet need for family planning, at 24% [2–4]. In 2018, the unmet need for family planning was 20% among married women in Zambia. The prevalence of unmet need for family planning in Zambia is above global and continental averages. This can be attributed to the slow rate of increase in the use of contraceptives. The modern contraceptive prevalence rate for women aged 15–49 in Zambia was reported to be at 48%, increasing from 33% in 2013–14 and 23% in 2001 [5, 6]. Unmet need for family planning can affect maternal and child health. Therefore, the potential health benefits of reducing unmet need for family planning are vast, as it would lead to a decline in unintended pregnancies leading to a reduction in maternal, infant and child mortality [7, 8]. It is estimated that satisfying unmet need for family planning alone could cut the number of maternal deaths by almost a third [2, 9].

From a reproductive health and a human rights point of view, all women of reproductive age should be able to have access to family planning methods to avoid unintended pregnancies and to space or limit the number of births. Fulfilling this right is an important intervention for improving maternal health, child health and the overall well-being of women and families. Following the 2012 London Summit on Family Planning, Zambia pledged to enhance its budgetary allocation to family planning commodities in order to eliminate unmet family planning needs and achieve universal family planning coverage by expanding the contraceptive technique mix and expanding access to underserved people [10, 11].

A scoping review study conducted in Ghana in 2019 to determine factors associated with unmet need for family planning among women of reproductive age in both low- and middle-income countries, revealed that age of a woman and a woman’s level of education were negatively associated with unmet need for family planning while the number of living children a woman had was found to be positively associated with unmet need for family planning [12, 13]. Among the reported reasons for non-use of contraceptives were opposition from husband or husband’s fear of infidelity, and a woman’s fear of side effects or other health concerns related to contraceptive methods [12, 13]. Literature on unmet need for family planning is extensive world-over, but there is little research that has been documented about its association with other individual level variables, such as women’s recent visits to a health facility, household wealth and exposure to mass media family planning messages in the sub-Saharan region and Zambia in particular.

The prevalence of unmet need for family planning provides information on the size of an important population sub-group for Family Planning (FP) programme management, that is, women at risk of pregnancy with a clear need for FP services based on their stated desire to space or limit births but are not using any family planning method [1, 2, 9, 14]. Successful implementation of the Family Planning 2020 initiative (FP2020) calls for targeted planning and resource investment in reproductive health interventions, with an expected production of positive health outcomes [1, 2, 9, 15, 16].

In Zambia, there are some studies on determinants of unmet need for family planning among married women using Demographic and Health Survey data. One study based on the 2007 Zambia Demographic and Health Survey data focused on variations in unmet need for contraception among ever married women. The study found that unmet need for limiting was associated with age at first marriage and a partner's desire for more children, while unmet need for spacing was associated with the number of children a woman has ever had and place of residence [13, 17–19]. Age and number of children a woman has ever had were strongly associated with unmet need for family planning.

Research has shown that unmet need for family planning can be associated with several factors, including; demographic, socio-economic, cultural and religious variables, as well as availability and accessibility of contraceptive methods. This study was conducted to examine the factors associated with unmet need for family planning among married women in Zambia, based on data from the recent Demographic and Health Survey. Results from this study will be useful in informing health policy direction and programming that targets to address challenges of meeting family planning needs for married women in Zambia.

## Methods

This study analysed data from the 2018 Zambia Demographic and Health Survey (ZDHS). The ZDHS is a cross-sectional nationally representative household survey which provides updated information on levels and trends in fertility, childhood mortality, family planning, maternal and child health indicators and HIV/AIDS for the country. It is carried out every four to five years and provides reliable health indicators at national, rural/urban and provincial levels.

The 2018 ZDHS used a stratified sample, selected in two stages from the 2010 Census of Population and Housing frame. In the first stage, 545 Enumeration Areas (EAs) were selected with probability proportional to the size of the enumeration area (EA). Household listing was then carried out in all the selected EAs and this provided a sampling frame for the second stage. In the second stage, 25 households were selected in each EA, with an equal probability systematic selection. Three questionnaires (household, woman and man) were used to collect data using face- to-face interviews conducted by trained enumerators [20]. The household questionnaire collected basic information about household members and household characteristics. All males aged 15–59 years and females in the age range of 15–49 years who were usual household members were eligible for an interview. Visitors who spent a night with the selected household were also eligible.

The target population in this study were currently married or in a union women aged 15–49 years. All women who were fecund and desired to wait for a minimum of two years before the subsequent birth or who did not want any more children were included in the study. Women who were sterilized or women whose husband/partner who was sterilized were excluded from the study. A total of 7,598 cases of married women were included in the analysis. The outcome variables for this study were current contraception use and unmet need for family planning which were recoded into binary outcomes. Contraception use was classified as: 1 = Using contraception and 0 = Not using a contraception method; and unmet need for family planning, was classified as; 0 = no unmet need and 1 = unmet need.

The explanatory variables included in the analysis were age, residence (urban/rural); parity, education, household wealth, employment status and visit to health facility in the 12 months prior to the survey. Other background variables included: exposure to media-based family planning messages and region/province which included all the ten provinces of Zambia.

Data were analysed using Stata software (SE) version 14.2. All analyses were weighted with the available survey weights to account for complex survey design. Descriptive statistics were used to present the background characteristics of the women included in the study. The *Chi*-square test of independence was used to determine the relationship between the outcome variables and the explanatory variables. Multivariate logistic regression analysis was applied to control for confounding and to determine the strength of association between the explanatory variables and outcome variables. Investigator-led backward stepwise regression analysis was used to develop a multivariable logistic regression model of predictors of unmet need for family planning in Zambia.

## Results

### Sample characteristics

A total of 7,598 married women aged 15–49 years were included in the analysis. Table 1 shows the percentage distribution of the study sample women according to selected background characteristics. The mean age for the married women was 28 years. Slightly over half (50.7%) of the married women had attended only primary education, about 35% had at least six live births. Results further show that knowledge of modern family planning method is almost universal among married women in Zambia (99.7%).

**Table 1:** Percent distribution of married women (15–49 years) by selected background characteristics, 2018 ZDHS

Background characteristics	Number	Percent
**Age**		
15–24	1875	24.5
25–34	2941	38.5
35–49	2832	37
**Residence**		
Urban	3080	40.3
Rural	4568	59.7
**Education level**		
No education	743	9.7
Primary	3881	50.7
Secondary	2635	34.5
Tertiary	389	5.1
**Children ever born**		
0–1	273	3.6
2–3	2486	32.5
4–5	2224	29.1
6+	2666	34.9
**Household wealth**		
Poor	3061	40
Medium	1468	19.2
Rich	3118	40.8
**Knowledge of family planning method**		
Knows no method or only traditional method	24	0.3
Knows any modern method	7624	99.7
**Visited health facility 12 months prior to survey**		
Yes	2286	29.9
No	5362	70.1
**Current employment status**		
Employed	4173	45.4
Not employed	3463	54.7
Total	7598	100

Table 2 shows the prevalence of unmet need for family planning and contraceptive use among currently married women included in our study by background characteristics. Bivariate analysis between contraceptive use and unmet need with the independent variables are presented to ascertain the association of socio-demographic variables on the outcomes of interest.

**Table 2:** Prevalence of contraceptive use and unmet need for family planning among married women (15–49) in Zambia, 2018 DHS

Background characteristics	Contraceptive use	p-value	Prevalence of Unmet need	p-value
	%		%	
**Age**		0.000***		0.0012*
15–24	46.2		19.8	
25–34	54.1		17.2	
35–49	47.2		22.2	
**Residence**		0.000***		0.003**
Rural	46.5		21.2	
Urban	54.3		17.4	
**Education level**		0.000***		0.000***
No education	37.7		24.2	
Primary	48.8		21.5	
Secondary	54.2		16.5	
Tertiary	49.6		14.8	
**Children Ever Born**		0.000***		0.000***
0–1	35.6		16.2	
2–3	53.9		15.9	
4–5	55.2		21.0	
6 +	49.6		26.0	
**Household Wealth index**		0.000***		0.000***
Poor	43.3		22.9	
Medium	53.0		18.2	
Rich	54.2		17.1	
**Employment Status**		0.1404^ns^		0.631^ns^
Employed	50.6		19.5	
Not employed	48.2		20.0	
Visited health facility in last 12 months		0.0025**		0.855^ns^
Yes	51.1		19.7	
No	46.0		19.5	
**Knowledge of family planning method**		0.000***		0.8926^ns^
Knows no method/knows only traditional method	2.5		20.8	
Know modern method	49.8		19.7	
Exposure to media FP messages		0.0285*		0.000***
No	48.8		20.8	
Yes	52.3		16.1	
Total	49.6		19.7	

***p < 0.001; **p < 0.01; *p < 0.05; ns = non-significant

The prevalence of unmet need for family planning among married women was 20%. The proportion of women with unmet need for family planning differs significantly between urban and rural areas, with rural areas having higher unmet need for family planning. There is an inverse relationship between educational attainment and unmet need for family planning, as married women with a lower educational attainment have a higher proportion of unmet need for family planning than women with secondary and tertiary levels of education. Married women who reported having six or more children ever born had a higher prevalence of unmet need for family planning compared to women with 3 or less children (26% *versus* 16%). Exposure to family planning messages among married women contributes to improvement in meeting women’s needs for family planning services.

Fifty percent of the married women in Zambia reported using any method of contraception in the 2018 ZDHS. Differences by region show that married women who live in urban areas are more likely to use contraception than women who reside in rural areas (54% *versus* 47%). It is also observed that married women who reported higher numbers of children ever born were more likely to use a contraception method. Married women who visited a health facility in the 12 months prior to the survey were also more likely to use contraception than women who did not visit the health facility. Furthermore, the study findings show that women who had exposure to media messages on family planning reported higher contraceptive use.

### Unmet need for family planning and associated factors

Results from the univariate logistic regression model showed that rural/urban residence, education, number of children ever born, wealth status, knowledge of family planning methods and exposure to media family planning messages were significantly associated with unmet need for family planning among married women in Zambia.

We also observed that, residence, education level, number of children ever born, wealth status, knowledge of family planning, visiting a health facility, exposure to media FP messages and region were the factors that were associated with contraceptive use among married women (Table 3).

**Table 3:** Univariate logistic regression analysis examining variations in contraceptive use and unmet need for family planning among married women age 15–49 in Zambia, DHS 2018

Background characteristics	Contraceptive use			Unmet need for family planning		
	OR	p-values	[95% CI]	OR	p-values	[95% CI]
**Age**						
15–24	1.00			1.00		
25–34	0.73	0.000	(0.633 0.836)	0.839	0.074	(0.691 1.018)
35–49	0.96	0.595	(0.828 1.114)	1.153	0.173	(0.939 1.415)
**Residence**						
Urban	1.00			1.00		
Rural	1.37	0.000	(1.202 1.561)	1.280	0.003	(1.090 1.502)
**Education level**						
No education	1.00			1.00		
Primary	0.635	0.000	(0.527 0.766)	0.856	0.162	(0.687 1.065)
Secondary	0.513	0.000	(0.419 0.627)	0.617	0.000	0.480 0.794)
Tertiary	0.627	0.001	(0.477 0.825)	0.542	0.002	(0.371 0.790)
**Children ever born**						
0–1	1.00			1.00		
2–3	0.472	0.000	(0.397 0.561)	0.976	0.832	(0.782 1.219)
4–5	0.451	0.000	(0.375 0.542)	1.369	0.003	(1.114 1.683)
6+	0.569	0.000	(0.482 0.672)	1.815	0.000	(1.476 2.231)
**Household wealth index**						
Poor	1.00			1.00		
Medium	0.677	0.000	(0.588 0.779)	0.748	0.002	(0.625 0.895)
Rich	0.644	0.000	(0.562 0.738)	0.695	0.000	(0.587 0.823)
**Current employment status**						
Not employed	1.00			1.00		
Employed	0.908	0.140	(0.799 1.032)	0.967	0.626	(0.843 1.109)
**Visited health facility in last 12 months**						
No	1.00			1.00		
Yes	0.815	0.003	(0.714 0.930)	1.017	0.855	(0.848 1.220)
**Knowledge of family planning method**						
Knows no method/knows only traditional method	1.00			1.00		
Know modern method	0.026	0.000	(0.003 0.191)	0.931	0.893	(0.328 2.638)
**Exposure to media FP messages**						
No	1.00			1.00		
Yes	0.869	0.029	(0.766 0.985)	0.733	0.000	(0.626 0.859)

***p < 0.001; **p < 0.01; *p < 0.05

### Determinants of unmet need for family planning 

In the multivariate model, results show that age, children ever born (parity), wealth status and exposure to media messages on family planning were significantly associated with unmet need for family planning among married women. The results are presented in Table 4.

**Table 4:** Multivariate regression analysis examining variations in contraceptive use and unmet need for family planning among married women age 15–49 in Zambia, DHS 2018

Background characteristics	Contraceptive use			Unmet need for family planning		
	AOR	p-values	[95% CI]	AOR	p-values	[95% CI]
**Age**						
15–24	1.000			1.000		
25–34	1.307	0.004	(1.087 1.571)	0.608	0.000	(0.471 0.784)
35–49	2.038	0.000	(1.703 2.621)	0.625	0.004	(0.453 0.863)
**Residence**						
Urban	1.000			1.000		
Rural	1.114	0.203	(0.943 1.316)	0.941	0.525	(0.781 1.134)
**Education level**						
No education	1.000			1.000		
Primary	0.641	0.000	(0,525 0.782)	0.929	0.459	(0.751 1.149)
Secondary	0.509	0.000	(0.399 0.650)	0.832	0.205	0.625 1.107)
Tertiary	0.559	0.001	(0.400 0.779)	0.907	0.654	(0.589 1.394)
**Children ever born**						
0–1	1.000			1.000		
2–3	0.397	0.000	(0.328 0.479)	1.188	0.041	(0.934 1.500)
4–5	0.278	0.000	(0.223 0.346)	1.918	0.000	(1.449 2.516)
6+	0.249	0.000	(0.196 0.316)	2.480	0.000	(1.799 3.486)
**Household wealth index**						
Poor	1.000			1.000		
Medium	0.726	0.000	(0.622 0.846)	0.781	0.007	(0.652 0.935)
Rich	0.730	0.001	(0.597 0.874)	0.856	0.226	(0.665 1.101)
**Current employment status**						
Employed	1.000			1.000		
Not employed	0.892	0.098	(0.779 1.021)	0.890	0.126	(0.766 1.033)
**Visited health facility in last 12 months**						
No	1.000			1.000		
Yes	0.811	0.002	(0.709 0.929)	0.992	0.08	(0.825 1.192)
**Knowledge of family planning method**						
Knows no method/knows only traditional method	1.000			1.000		
Know modern method	0.536	0.004	(0.008 0.381)	1.110	0.839	(0.406 3.030)
**Exposure to media FP messages**						
No	1.000			1.000		
Yes	1.017	0.807	(0.890 1.160)	0.820	0.018	(0.695 0.967)

***p < 0.001; **p < 0.01; *p < 0.05

Our study suggests that increasing age is associated with a progressive reduction in unmet need. Women in the age groups 25–34 and 35–49 were less likely to have unmet need for family planning compared to women aged 15–24 years {(AOR = 0.61; 95% CI 0.47, 0.78; p < 0.001) and (AOR = 0.63; 95% CI 0.45, 0.86; p = 0.004), respectively}. Women with 4–5 births were about twice as likely to have an unmet need for family planning compared to women with 0–1 births (AOR = 1.90; 95% CI 1.45, 2.50; p < 0.001). Women who had six births or more had highest odds of unmet need for family planning when compared with women who had 0–1 births (AOR = 2.48; 95% CI 1.80, 3.49; p < 0.001).

Our study also found that women who were exposed to family planning messages on television, radio or newspapers were 18% less likely to have unmet need for FP, relative to women who were not exposed (AOR = 0.82; 95% CI 0.70, 0.97; p = 0.018). Women from medium wealth households had reduced chances of unmet need for family planning compared to women who belonged to poor households (AOR = 0.78; 95% CI 0.65, 0.94; p = 0.007).

## Discussion

Our study found that age, children ever born (parity), household wealth and exposure to media-based family planning messages are significantly associated with unmet need for family planning among married women. The low use of contraception and the unmet need for family planning among married women are a public health concern in the country. These results suggest urgent attention for strengthening access to family planning information and also shows the need to address socio-demographic factors associated with unmet need for family planning. Conversely, the study has revealed that education, visits to health facilities 12 months prior to the survey and knowledge of a family planning modern method had no effect on unmet need for family planning.

The age of a woman plays a significant role in determining the extent of unmet need for family planning. Our study found that a woman’s age was negatively associated with unmet need, implying that as a woman gets older, the unmet need decreases. Unmet need is lower among the older age women, probably because these women may have achieved their desired family size. This finding is confirmed by related studies conducted in Ethiopia and Ghana, that found that as the woman’s age increased by one year, the odds of having unmet need were less by 20% [3, 7, 13, 21].

An increase in the number of births a married woman has ever had, is associated with an increase in unmet need. These results are similar to what was found in a study conducted in 2019 in Ethiopia. The study found that as parity increased by one birth, the chance of having unmet need for family planning was twice as much [7, 8, 21]. In another related study conducted in Burkina Faso in 2014, women who had at least five living children, were approximately eight times likely to have an unmet need for contraception than those who did not have living children [8, 22].

The findings from our study suggest that there were no differences in proportions of unmet need for family planning by education level. Education may not have an impact on unmet need for family planning but rather, targeted family planning information. Our findings show that a married woman's exposure to mass media-disseminated family planning messages was strongly linked to a lower risk of unmet need. This outcome validates the findings from previous studies conducted in other African countries [23–25]. The studies concluded that women who are exposed to family planning messages through radio or television are more protected against unmet need. It may be argued that the media has the power to raise family planning awareness among married women. A woman’s exposure to media-based family planning messages enhances her awareness and knowledge on the benefits of family planning services. Met need for family planning has a potential to significantly reduce unwanted pregnancies, abortions and limit family size, as well as avoid child and maternal deaths [1, 2, 9, 26].

Visiting a health facility presents an opportunity for a woman to get information on health matters, access to counselling services, and also access to family planning services. It is expected that women who access health facilities may have a high likelihood of met need for family planning. Studies conducted in Ghana and Burundi established that women who visited a heath facility had reduced odds of unmet need [13, 23]. However, findings in this study indicate that the prevalence of unmet need for FP is not significantly associated with visiting a health facility during the twelve months preceding the 2018 ZDHS. This may be because routine family planning services at government health facilities in the country are provided for very limited hours and are not coordinated with other services that women attend, such as child immunisation clinics [22, 27, 28].

## Conclusion

A high proportion of married women have an unmet need for family planning and this is significantly associated with age, children ever born (parity), household wealth and exposure to media messages on family planning. Based on the findings, there is need to enhance access to family planning information and programming targeted towards younger women. Further, optimizing media-based sensitisation campaigns on the benefits of using contraceptives may have greater impact on uptake and on reducing unplanned pregnancies. Ensuring availability of a wide range of family planning methods for women to choose from, could potentially improve uptake and reduce unmet need for family planning. Addressing underlying structural factors that improve the wealth status of women may contribute to improving unmet need for family planning among married women.

## Abbreviations

AOR: Adjusted odds ratio
CI: Confidence interval
DHS: Demographic and Health Survey
EA: Enumeration Area
FP: Family planning
OR: Odds ratio
UNFPA: United Nations Population Fund
UN: United Nations
UOR: Unadjusted odds ratio
WHO: World Health Organisation
ZamStats: Zambia Statistics Agency
ZDHS: Zambia Demographic and Health Survey

## Declarations

### Availability of data and materials

Data used in the study is publicly available on the DHS Program Website (https://dhsprogram.com/). Other materials such as do-files can be provided upon request from the corresponding author (harrietnamukoko@yahoo.com).
